# A Wet Gas Metering System Based on the Extended-Throat Venturi Tube

**DOI:** 10.3390/s21062120

**Published:** 2021-03-17

**Authors:** Haobai Xue, Peining Yu, Maomao Zhang, Haifeng Zhang, Encheng Wang, Guozhu Wu, Yi Li, Xiangyuan Zheng

**Affiliations:** 1 Tsinghua Shenzhen International Graduate School, Tsinghua University, Shenzhen 518055, China; xue.haobai@sz.tsinghua.edu.cn (H.X.); liyi@sz.tsinghua.edu.cn (Y.L.); zheng.xiangyuan@sz.tsinghua.edu.cn (X.Z.); 2 Shenzhen Institute of Information Technology, Shenzhen 518172, China; peining.yu@sziit.edu.cn; 3 Research Institute of Tsinghua, Pearl River Delta, Guangzhou 510700, China; zhanghf@tsinghua-gd.org; 4 Wenliu Oil Production Plant of Zhongyuan Oilfield, SINOPEC, Puyang 457001, China; wangec.zyyt@sinopec.com; 5 Shenzhen LeEngSTAR Technology Co., Ltd., Shenzhen 518055, China; wuguozhu@leengstar.com

**Keywords:** extended-throat Venturi tube, ETV, classical Venturi tube, gas over-reading, wet gas metering, iteration algorithm, relative error distribution

## Abstract

Although the use of a classical Venturi tube for wet gas metering has been extensively studied in the literature, the use of an extended-throat Venturi (ETV) tube has rarely been reported since its first proposal by J. R. Fincke in 1999. The structure of an ETV is very simple, but due to the complexity of multiphase flow, its theoretical model has not been fully established yet. Therefore, in this paper theoretical models have been developed for the convergent and throat sections of an ETV, and the gradients of front and rear differential pressures are derived analytically. Several flowrate algorithms have been proposed and compared with the existing ones. Among them, the iteration algorithm is found to be the best. A reasonable explanation is provided for its performance. The relationship between the differential pressure gradient and the flowrate relative error is also studied, such that the relative error distributions varying with ETV measured flowrates can be derived. The gas flowrate error of ETV increases with the liquid content whilst the liquid flowrate error of ETV decreases with the liquid content, and the relative errors of liquid flowrate are generally 2 to 3 times larger than that of the gas flowrate. Finally, the ETV tends to be more accurate than the classical Venturi tube. The ETV can be designed more compact under the same signal intensity due to its significantly higher velocity in the throat section.

## 1. Introduction

**Figure 1:**
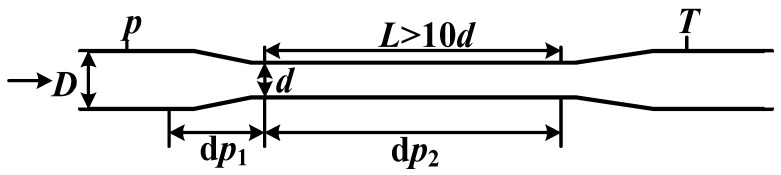
Schematic diagram of an extended-throat Venturi (ETV) tube.

Wet gas typically refers to a type of two-phase flow with the gas volume fraction (GVF) larger than 95% or the Lockhard Martinelli (L-M) number less than 0.3. Compared with the oil–gas–water multiphase flow, the flow pattern and governing rule of wet gas are relatively stable and simple, and therefore, there are many companies dedicated to wet gas metering and the most commonly employed device is the classical Venturi tube. The structure and algorithm of a classical Venturi tube for single-phase metering are well known and included in the ISO 5167-4 [1], and its algorithms for wet gas metering have been extensively studied in the literature and finally included in the ISO/TR 11583 [2]. In contrast, the study of an extended-throat Venturi (ETV) tube for wet gas metering is rarely reported in the literature since its first proposal by J. R. Fincke [3,4]. As shown in Figure 1, the basic structure of an ETV tube is very simple. It includes a convergent section for front differential pressure $dp_{1}$ measurement and a throat section for rear differential pressure $dp_{2}$ measurement, as well as a pressure sensor at the inlet and a temperature sensor at the outlet for measuring p and T respectively. The gas and liquid flowrate can be calculated from the above four variables and a direct fitting method has been proposed by Fincke himself [3,4]. Thereafter, multiple variables nonlinear regression [5,6], artificial neural network (ANN), support vector machine (SVM) [7,8] and fluctuating-property-based (FPB) [9,10] methods have been employed to increase its accuracy. However, despite its simple structure, its theoretical model and working principle have not been fully established and explained yet. For example, both the homogeneous flow model [7,8] and separated-flow model [9,10] have been established for the throat section of ETV, but no matter the gas-liquid flow is ideally mixed or separated, there will be no difference between the convergent and throat sections to be exploited for gas/liquid flowrate measurement. Methods such as ANN, SVM, and FPM tend to require large numbers of reference data as the training set, which are usually difficult and expensive to acquire in the oil fields, and their ability to extrapolate beyond their test range still need to be proved. Therefore, practical algorithms of ETV for wet-gas metering are largely lacking in the open literature.

In recent years, other measuring techniques, such as electrical capacitance [11,12], photon (gamma and X-ray) attenuation [13] and ultrasound [14,15], have been increasingly used in ETV for determining the void fraction and flow regimes of multiphase flow. For example, a wet-gas flowmeter based on an ETV tube and a sonar flowmeter has been proposed by Weatherford [16,17]. It uses the sonar flowmeter to determine the convection velocity and thus the volumetric flowrate. Another example is a multiphase flowmeter that combines an ETV with two microwave resonators [18]. The time-based responses of these two sensors are correlated to extract the average flow velocity and the volumetric flowrate. These two examples essentially exploited the difference between a differential pressure (DP) flowmeter and a velocity (volumetric) flowmeter to yield the gas and liquid flowrates, which is different from the conventional ETV that uses the difference between two DP flowmeters for flowrate measurement. Therefore, these types of ETV are beyond the scope of this paper and only conventional ETV will be discussed in the following parts.

In this paper, the theoretical models of the convergent and throat section of an ETV tube are established, and the working principle of the wet gas metering system is explained. Thereafter, several flowrate algorithms based on direct fitting method and iteration method are proposed and compared with the existing ones. It is found that the iteration method enjoys higher prediction accuracy than the linear fitting method, and a reasonable explanation is provided. Finally, the gradients of the front differential pressure (∂d*p*_1_/∂*Q*_g_, ∂d*p*_1_/∂*Q*_l_) and rear differential pressure (∂d*p*_2_/∂*Q*_g_, ∂d*p*_2_/∂*Q*_l_) are derived, and their relationships with the relative errors of gas and liquid flowrate are studied. As a result, the error distribution maps of an ETV are obtained from the test results, and these maps are compared with the ones of a classical Venturi tube found in the literature. The results show that the ETV tends to have higher measurement accuracy than the classical Venturi tube. In addition, the ETV can be designed to be more compact due to its significantly higher flow velocity in the throat section.

## 2. Theoretical Model

### 2.1. Convergent Section

When pure gas flows through an ETV tube, and the measured front differential pressure is $dp_{1}$, then its the volume flowrate can be directly calculated by:

(1) $$Q_{go}=\frac{C_{d}\varepsilon }{\sqrt{1-\beta^{4}}}A\sqrt{\frac{2\Delta p_{1}}{\rho_{g}}}$$

where β=d/D, $A=\pi d^{2}/4$, $C_{d}$ is the discharge coefficient, ε is the expansion factor, and the values of $C_{d}$ and ε can be determined from ISO 5167-4 [1]. $\rho_{g}$ denotes the gas density, which can be calculated from the measured pressure p and temperature T according to ISO 12213-2 [19].

However, when wet gas flows through an ETV, the flowrate obtained from Equation (1) is referred to as the “indicated” gas flowrate $Q_{tp}$, and the “actual” gas flowrate $Q_{g}$ should be calculated by $Q_{g}=Q_{tp}/\phi_{g}$, where $\phi_{g}$ is referred to as the “gas over-reading”. $\phi_{g}$ is always larger than one because the front differential pressure $dp_{1}$ of wet gas is contributed by the gas and liquid together.

$\phi_{g}$ is usually calculated from the Lockhard–Martinelli number X, and the equation employed is referred to as the over-reading equation. For example, Murdock [20], Bizon [21], Lin et al. [22] proposed a linear model like:

(2) $$\phi_{g1}=a_{1}X+b_{1}$$

where $X=\frac{Q_{l}}{Q_{g}}\sqrt{\frac{\rho_{l}}{\rho_{g}}}$, whereas Chisholm [23], de Leeuw [24], Reader-Harris [25] proposed a general model like:

(3) $$\phi_{g1}=\sqrt{1+C_{1}X+X^{2}}$$

where:

(4) $$C_{1}=S_{1}\sqrt{\frac{\rho_{g}}{\rho_{l}}}+\frac{1}{S_{1}}\sqrt{\frac{\rho_{l}}{\rho_{g}}}={\left(\frac{\rho_{g}}{\rho_{l}}\right)}^{n}+{\left(\frac{\rho_{l}}{\rho_{g}}\right)}^{n}$$

where $S_{1}=u_{g}/u_{l}$ is referred to as the slip ratio. Equations (3) and (4) are referred to as the general model because its basic form can be theoretically derived. For example, if the pressure drop of convergent section is assumed to be dominated by the accelerate pressure drop [26], then the differential form of the pressure drop can be written as:

(5) $$-Adp_{tp}=d\left[\frac{\rho_{g}Q_{g}^{2}}{A\alpha }+\frac{\rho_{l}Q_{l}^{2}}{A\left(1-\alpha \right)}\right]$$

By assuming the cross-sectional void fraction α as a constant and integrating Equation (5) from the Venturi inlet to the throat, we have:

(6) $$dp_{1}=\frac{1}{2}\frac{1-\beta^{4}}{A^{2}}\left[\frac{\rho_{g}Q_{g}^{2}}{\alpha }+\frac{\rho_{l}Q_{l}^{2}}{\left(1-\alpha \right)}\right]$$

By using similar method for gas and liquid single-phase, we have:

(7) $$dp_{1}=\frac{1}{2}\frac{1-\beta^{4}}{A^{2}}\frac{\rho_{g}Q_{g}^{2}}{\alpha^{2}}=\frac{1}{2}\frac{1-\beta^{4}}{A^{2}}\frac{\rho_{l}Q_{l}^{2}}{{\left(1-\alpha \right)}^{2}}$$

From Equations (6) and (1), it can be derived that the gas over-reading is in the form of Equations (3) and (4). Meanwhile, it can be derived from Equation (7) that $S_{1}=\sqrt{\frac{\rho_{l}}{\rho_{g}}}$ for this scenario, and this model is referred to as the stratified model as no friction force is assumed between the gas and liquid. On the other hand, if the force between gas and liquid is very strong so that no velocity slip exists, which means $S_{1}=1$, then this model is referred to as the homogeneous model. The stratified and homogeneous model can be viewed as two extreme scenarios of $S_{1}$, therefore, $S_{1}$ usually varies between 1 and $\sqrt{\frac{\rho_{l}}{\rho_{g}}}$ in actual working conditions, and its exact value is also dependent on the gas Froude number $Fr_{g}$, the types of fluids and the structure parameter of a Venturi tube (e.g., diameter ratio β).

If the Equation (1) and the definitions of X and $\phi_{g}$ are substituted into Equation (3) and simplified, then the following expression can be obtained:

(8) $$2K_{1}^{2}dp_{1}=Fr_{g}^{2}+\left(\frac{1}{S_{1}}\sqrt{\frac{\rho_{l}}{\rho_{g}}}+S_{1}\sqrt{\frac{\rho_{g}}{\rho_{l}}}\right)Fr_{g}Fr_{l}+Fr_{l}^{2}$$

where $Fr_{g}$ is the gas Froude number, $Fr_{g}=\frac{Q_{g}}{A_{D}\sqrt{gD}}\sqrt{\frac{\rho_{g}}{\rho_{l}-\rho_{g}}}$; $Fr_{l}$ is the liquid Froude number, $Fr_{l}=\frac{Q_{l}}{A_{D}\sqrt{gD}}\sqrt{\frac{\rho_{l}}{\rho_{l}-\rho_{g}}}$, $K_{1}=\frac{C_{d}\varepsilon }{\sqrt{1-\beta^{4}}}\frac{\beta^{2}}{\sqrt{gD\left(\rho_{l}-\rho_{g}\right)}}$ and $A_{D}=\pi D^{2}/4$. It is worth mentioning that $X=Fr_{l}/Fr_{g}$.

Therefore, the gradient of the front differential pressure $dp_{1}$ can be written as:

(9) $$\nabla \left(dp_{1}\right)=\frac{Fr_{g}}{2K_{1}^{2}}\left[\begin{array}{c} 2+\left(\frac{1}{S_{1}}\sqrt{\frac{\rho_{l}}{\rho_{g}}}+S_{1}\sqrt{\frac{\rho_{g}}{\rho_{l}}}\right)X\\ \left(\frac{1}{S_{1}}\sqrt{\frac{\rho_{l}}{\rho_{g}}}+S_{1}\sqrt{\frac{\rho_{g}}{\rho_{l}}}\right)+2X \end{array}\right]$$

From Equation (9) it can be noted that the gradient of $dp_{1}$ is a function of the slip ratio $S_{1}$, the density ratio $\sqrt{\frac{\rho_{l}}{\rho_{g}}}$ and the Lockhard-Martinelli number X. Its direction is not affected by the gas Froude number $Fr_{g}$ and the parameter $K_{1}$.

### 2.2. Throat Section

When pure gas flows through a throat tube with a diameter of d and length of L, its pressure drop can be calculated by the Fanning’s law of friction:

(10) $$\Delta p_{go}=\frac{\tau_{wg}P_{go}L}{A}=\lambda \frac{L}{d}\frac{\rho_{g}}{2}{\left(\frac{Q_{g}}{A}\right)}^{2}$$

where $\tau_{wg}$ is the shear stress of gas on wall, $\tau_{wg}=\frac{\lambda \rho_{g}u_{go}^{2}}{8}$; $P_{go}$ is the perimeter of gas at pipe wall, $P_{go}=\pi d$ for pure gas; A is the cross-sectional area of the throat, $A=\frac{\pi d^{2}}{4}$. λ is the pipe friction factor, if the pipe wall is rough and flow is turbulent, then λ is a constant which does not change with the Reynold number Re.

After some modifications of Equation (10), the indicated gas flowrate of the throat section can be obtained:

(11) $$Q_{tp2}=\frac{1}{\sqrt{\lambda L/d}}A\sqrt{\frac{2\Delta p_{2}}{\rho_{g}}}$$

When wet gas flows through the same throat tube, the pressure drop of liquid and gas can be derived from the momentum conservation equation [23]:

(12) $$-A_{l}\frac{dp_{tp}}{dz}=\tau_{wl}P_{l}-S_{F}$$

for liquid, and:

(13) $$-A_{g}\frac{dp_{tp}}{dz}=\tau_{wg}P_{g}+S_{F}$$

for gas, where $S_{F}$ is the shear force per unit length at the interface between the phases.

If the pipe friction factor λ and the hydraulic diameter $\chi =4P_{i}/A_{i}$ are assumed the same for the gas and liquid, then divide Equation (12) by Equation (13), we have:

(14) $$Z^{2}=\frac{-A_{l}\frac{dp_{2}}{dz}+S_{F}}{-A_{g}\frac{dp_{2}}{dz}-S_{F}}=\frac{\rho_{l}}{\rho_{g}}{\left(\frac{u_{l}}{u_{g}}\right)}^{2}$$

After substituting Equation (14) into Equation (13) and cancelling $S_{F}$, we have:

(15) $$-\frac{dp_{tp}}{dz}=\lambda \frac{L}{d}\frac{\rho_{g}}{2}{\left(\frac{Q_{g}}{A_{g}}\right)}^{2}\frac{Z^{2}+\frac{A_{g}}{A_{l}}}{\frac{A_{g}}{A_{l}}+1}$$

From Equations (15) and (10), it can be derived that:

(16) $$\phi_{g}^{2}=1+\left(Z+\frac{1}{Z}\right)X+X^{2}$$

where $Z=\frac{1}{S_{2}}\sqrt{\frac{\rho_{l}}{\rho_{g}}}$, as shown in Equation (14). The expression of Equation (16) for the throat tube is identical with Equation (3) for the convergent one, but it is generally believed that the flow within it is more or less homogenous, so that $S_{2}\approx 1$ [27].

Similar to the convergent section, the throat section also has its own linear model as Equation (2) and general model as Equation (3), and the gradient of rear differential pressure $dp_{2}$ can also be written as similar form as Equation (9):

(17) $$\nabla \left(dp_{2}\right)=\frac{Fr_{g}}{2K_{2}^{2}}\left[\begin{array}{c} 2+\left(\frac{1}{S_{2}}\sqrt{\frac{\rho_{l}}{\rho_{g}}}+S_{2}\sqrt{\frac{\rho_{g}}{\rho_{l}}}\right)X\\ \left(\frac{1}{S_{2}}\sqrt{\frac{\rho_{l}}{\rho_{g}}}+S_{2}\sqrt{\frac{\rho_{g}}{\rho_{l}}}\right)+2X \end{array}\right]$$

where $K_{2}=\frac{1}{\sqrt{\lambda L/d}}\frac{\beta^{2}}{\sqrt{gD\left(\rho_{l}-\rho_{g}\right)}}$.

### 2.3. Working Principle

By comparing Equations (9) and (17), it can be noted that for the ETV tube, the L-M number X and density ratio $\sqrt{\frac{\rho_{l}}{\rho_{g}}}$ of the convergent section and the throat section are always the same, therefore, the only possible reason for the intersection angle between $\nabla \left(dp_{1}\right)$ and $\nabla \left(dp_{2}\right)$ is the difference in slip ratio S. Some scholars believe that the flow in the throat section is very close to the homogeneous flow (e.g., $S_{2}=1$ or $n_{2}=0.5$) [27], whilst the flow in the convergent section may have a higher slip ratio $S_{1}$ due to the existence of the acceleration pressure drop (e.g., $S_{1}>1$, or $n_{1}<0.5$), and the ETV uses this difference to calculate the gas and liquid flowrates.

**Figure 2:**
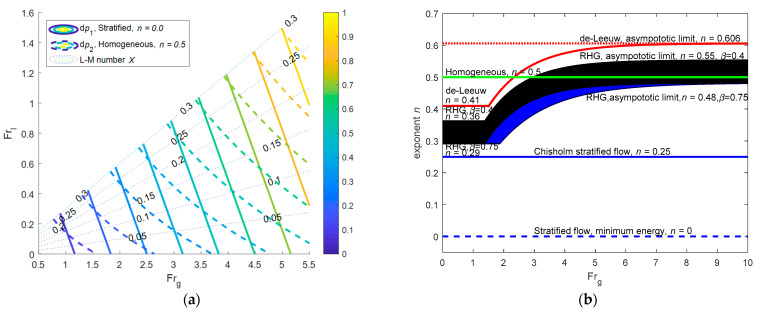
Schematic diagram of the working principle: (**a**) the contours of $dp_{1}$ and $dp_{2}$ in the ideal case; (**b**) the variations of index n of several common over-reading models in the real case. (The filled area corresponds to the Reader-Harris model under β=0.4~0.75, where the black area represents the oil whilst the blue one represents the water. n=0.5 corresponds to the homogenous model, n=0.25 corresponds to the Chisholm orifice plate model, whereas n=0 corresponds to the stratified model or dense phase model ($\rho_{g}=\rho_{l}$)) [28].

In the ideal case, if there is only acceleration pressure drop in the convergent section whilst there is only frictional pressure drop in the throat section, then the contours of the non-dimensional front differential pressure $dp_{1}$ and rear differential pressure $dp_{2}$ are shown in Figure 2a. From Figure 2a, it can be noted that there is always an intersection angle between $\nabla \left(dp_{1}\right)$ and $\nabla \left(dp_{2}\right)$ and the intersection angle varies with X, as shown in Equations (9) and (17). In the real case, the index n of Equation (3) is obtained from data fitting [28]. The variations of index n of several common over-reading models with gas Froude number $Fr_{g}$ are shown in Figure 2b [28], from which it can be noted that the index n is also affected by the fluid type and diameter ratio β. Therefore, it will be difficult to continue using the analytical method for the derivation of index n.

## 3. Flowrate Algorithms

**Figure 3:**
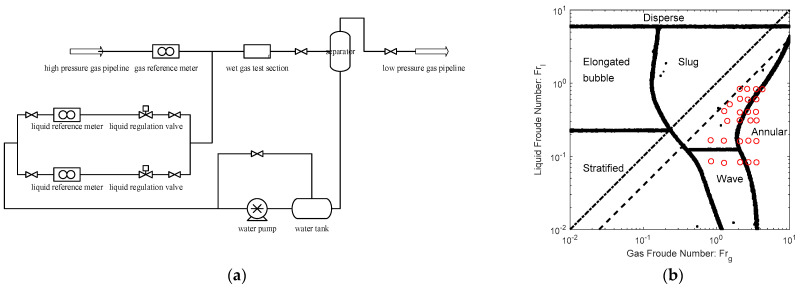
Schematic diagram of the multiphase flow experimental facility and test data: (**a**) experimental facility; (**b**) test data.

The experiments of ETV were carried out at the wet gas test facility of Chengdu Verification Branch of National Oil and Gas Large Flowrate Measurement Station of China (CVB) which is designed for gas-liquid two phase flow studies consisting of water and natural gas. The schematic diagram of the CVB wet gas test facility is shown in Figure 3a, from which it can be noted that this facility is mainly composed of the gas and liquid reference meters, the liquid injection and regulation system and the gas-liquid separation system. The gas used for the wet gas experiments comes from a high pressure pipeline, and is metered by a high accuracy ultrasonic flowmeter before mixing. Then the natural gas flows into the test section where the liquid is injected and mixed. The flowrate of the liquid is regulated by the liquid injection and regulation system. A 0.5 inch and a 1 inch Coriolis mass flow meters work as liquid reference meters on the liquid injection line. The natural gas drives the liquid through the test section and then flow into the gas-liquid separation system where the gas and liquid are separated. The separated liquid is returned to the water tank and recirculated by the liquid pump, whereas the separated natural gas is discharged into a low-pressure pipeline. The test pressure range of the CVB is from 1.5 to 4.0 MPa, the gas flow test range is from 8 m^3^/h to 650 m^3^/h, and the liquid flow test range is from 0 to 8 m^3^/h. The CVB is a Class A metering station with a measurement uncertainty (k=2) as low as 0.05%~0.07%, and more relevant information can be found in [29,30].

In order to simulate the flow conditions of the Yakela gas fields in northwestern China, the whole system is pressurized to 2.0 MPa, and the gas and liquid flowrate ranges are 32~156 m^3^/h and 0~4.1 m^3^/h respectively. There are 31 test points in total, where 5 of them represent the pure gas working conditions, whilst the rest 26 points represent the wet gas ones and their distribution are shown in Figure 3b, where the dash-dotted line represents the Lockhart–Martinelli (L-M) number of 1.0 whereas the dashed line represents the L-M number of 0.4. Therefore, it is notable that the L-M range of the test points is 0~0.4, and the flow patterns are mainly annular and slug flows.

All of the measured ETV have the same diameter ratio of β=d/D=0.5, whereas their inlet diameters D can be 50 or 80 mm to satisfy the requirements of different flowrate ranges. As the effect of D on the flowrate algorithm is negligible, only the test results of D=50 mm are shown below to avoid redundancy. The detection frequency of the ETV tube is f=10 Hz.

For each measurement, there is an associated error. Therefore, it is sometimes more convenient to use mean absolute error (MAE) or mean absolute percentage error (MAPE) to compare the average values of errors of different data sets. The definitions of MAE and MAPE are as follows:

(18) $$\mathrm{MAE}=\frac{1}{n}\overset{n}{\underset{i=1}{\sum }}\left|{\hat{y}}_{i}-y_{i}\right|$$

(19) $$\mathrm{MAPE}=\frac{100\%}{n}\overset{n}{\underset{i=1}{\sum }}\left|\frac{{\hat{y}}_{i}-y_{i}}{y_{i}}\right|$$

where ${\hat{y}}_{i}$ is the measured value, $y_{i}$ is the reference value and n is the number of measurements of a dataset. The ranges of MAE of MAPE are both from 0 to infinity and larger MAE and MAPE mean lower accuracy. However, MAE is usually used for the errors of ratios (e.g., $\phi_{g}$ and $Y/Y_{max}$) whereas MAPE is mainly used for the errors of flowrates (e.g., $Q_{g}$ and $Q_{l}$).

### 3.1. Direct Fitting Method

#### 3.1.1. Linear Model

Simultaneous solution of the linear over-reading Equation (2) of the convergent and throat sections leads to the linear models as follows:

(20) $$\left[\begin{array}{c} Q_{gt1}\\ Q_{gt2} \end{array}\right]=\left[\begin{array}{cc} a_{1}\sqrt{\frac{\rho_{l}}{\rho_{g}}} & b_{1}\\ a_{2}\sqrt{\frac{\rho_{l}}{\rho_{g}}} & b_{2} \end{array}\right]\left[\begin{array}{c} Q_{l}\\ Q_{g} \end{array}\right]$$

Matrix inversion can be applied to Equation (20) directly and the result is:

(21) $$\left[\begin{array}{c} Q_{l}\\ Q_{g} \end{array}\right]=\left[\begin{array}{cc} a_{1}^{*}\sqrt{\frac{\rho_{g}}{\rho_{l}}} & b_{1}^{*}\sqrt{\frac{\rho_{g}}{\rho_{l}}}\\ a_{2}^{*} & b_{2}^{*} \end{array}\right]\left[\begin{array}{c} Q_{gt1}\\ Q_{gt2} \end{array}\right]$$

where $a_{1}^{*}=\frac{b_{2}}{a_{1}b_{2}-a_{2}b_{1}}$, $a_{2}^{*}=\frac{-a_{2}}{a_{1}b_{2}-a_{2}b_{1}}$, $b_{1}^{*}=\frac{-b_{1}}{a_{1}b_{2}-a_{2}b_{1}}$, $b_{2}^{*}=\frac{a_{1}}{a_{1}b_{2}-a_{2}b_{1}}$.

However, it is worth mentioning that the matrix of Equation (20) is relatively ill conditioned, so large errors may be generated during the matrix inversion. Therefore, variable substitution method may be used instead and the gas and liquid flowrates are directly fitted with the indicated gas flowrate $Q_{gt1}$ and $Q_{gt2}$, so as to prevent this matrix inversion process.

The linear model is easy to solve but has a limited application range, so it is usually used together with the classification method. However, as the linear model enjoys the merits of simplicity and stability, it can be used for: (1) providing initial values for iteration; (2) providing reference for classification (such as estimating the value of $Fr_{g}$); (3) fix the “bad values” or “blind spots” of some complicated algorithms.

#### 3.1.2. General Model

Reader–Harris and Graham improves the conventional Chisholm model [25]. They believe in the gas-liquid two-phase flow, the discharge coefficient of the Equation (1) changes, so they added a correction factor before the Chisholm model to achieve better data fitting results [25]:

(22) $$\phi_{g1}=C_{3}\sqrt{1+C_{1}X+X^{2}}$$

(23) $$\phi_{g2}=C_{4}\sqrt{1+C_{2}X+X^{2}}$$

By solving the above two equations simultaneously, we can get:

(24) $$Q_{g}=\sqrt{a_{1}Q_{gtp1}^{2}+a_{2}Q_{gtp2}^{2}\pm \sqrt{{\left(a_{1}Q_{gtp1}^{2}+a_{2}Q_{gtp2}^{2}\right)}^{2}-{\left(a_{3}Q_{gtp1}^{2}+a_{4}Q_{gtp2}^{2}\right)}^{2}}}$$

(25) $$Q_{l}=\sqrt{a_{1}Q_{ltp1}^{2}+a_{2}Q_{ltp2}^{2}\pm \sqrt{{\left(a_{1}Q_{ltp1}^{2}+a_{2}Q_{ltp2}^{2}\right)}^{2}-{\left(a_{3}Q_{ltp1}^{2}+a_{4}Q_{ltp2}^{2}\right)}^{2}}}$$

where $a_{1}=\frac{-C_{2}}{2C_{3}^{2}\left(C_{1}-C_{2}\right)}$, $a_{2}=\frac{C_{1}}{2C_{4}^{2}\left(C_{1}-C_{2}\right)}$, $a_{3}=\frac{1}{C_{3}^{2}\left(C_{1}-C_{2}\right)}$, $a_{4}=\frac{-1}{C_{4}^{2}\left(C_{1}-C_{2}\right)}$.

Similar to the linear model, variable substitutions can also be implemented to prevent the errors introduced during the solving process.

### 3.2. Iteration Method

#### 3.2.1. Over-Reading Equations

At present, there are a large number of over-reading equations related to wet gas in the literature. Solartron ISA summarizes this [31,32], and most of the common over-reading equations can be written as the form of Equations (3) and (4). This equation is also referred to as the general model as its basic form can be derived theoretically, but the index n is usually determined experimentally and it has become the key part for a good over-reading model. Currently, the most common over-reading models for wet gas measurement includes:

1. Homogeneous Model

The homogeneous model assumes the gas and liquid are uniformly mixed without any slip, so the wet gas is treated as an pseudo-single phase, and the constant $C_{1}$ in Equations (3) and (4) becomes:

(26) $$C_{\mathrm{Hom}}=\sqrt{\frac{\rho_{g}}{\rho_{l}}}+\sqrt{\frac{\rho_{l}}{\rho_{g}}}$$

So n=0.5 and it is notable that the index n of homogeneous model is a constant. Therefore, if the L-M number is known, this model can obtain ${\hat{\phi }}_{g}$ directly and then estimate the gas/liquid flowrate. In this paper, the homogeneous model will be used for providing the initial values for other more complicated models.

2. de Leeuw Model

de Leeuw tested a Venturi tube with an inner diameter of four inches and β=0.4 under different pressures with nitrogen and diesel oil as the flow medium. It is found that the index n varies between 0.41 and 0.606, and its specific form is:

(27) $$n=\{\begin{array}{cc} 0.606\left(1-e^{-0.746Fr_{g}}\right) & \mathrm{if}\;Fr_{g}\geq 1.5\\ 0.41 & \mathrm{if}\;0.5\leq Fr_{g}<1.5 \end{array}$$

It is notable that the de Leeuw model needs to know $Fr_{g}$ first and calculates n and $C_{1}$ latter, so ${\hat{\phi }}_{g}$ should be determined through an iteration method.

3. Reader-Harris Model

Reader-Harris conducted a series of experiments for a Venturi tube with 0.4≤β≤0.75, and obtained the index n as follows:

(28) $$n=\max\left[\begin{array}{cc} 0.583-0.18\beta^{2}-0.578\exp\left(\frac{-0.8Fr_{g}}{H}\right), & 0.392-0.18\beta^{2} \end{array}\right]$$

where H depends on the liquid types, which is 1.0 for hydrocarbon liquid, 1.35 for water at ambient and 0.79 for liquid water in a wet-steam flow.

In addition, the discharge coefficient is also changed as follows:

(29) $$C_{d}=1-0.0463\exp\left(-0.05Fr_{g}\right)\min\left(\begin{array}{cc} 1, & \sqrt{\frac{X}{0.016}} \end{array}\right)$$

**Figure 4:**
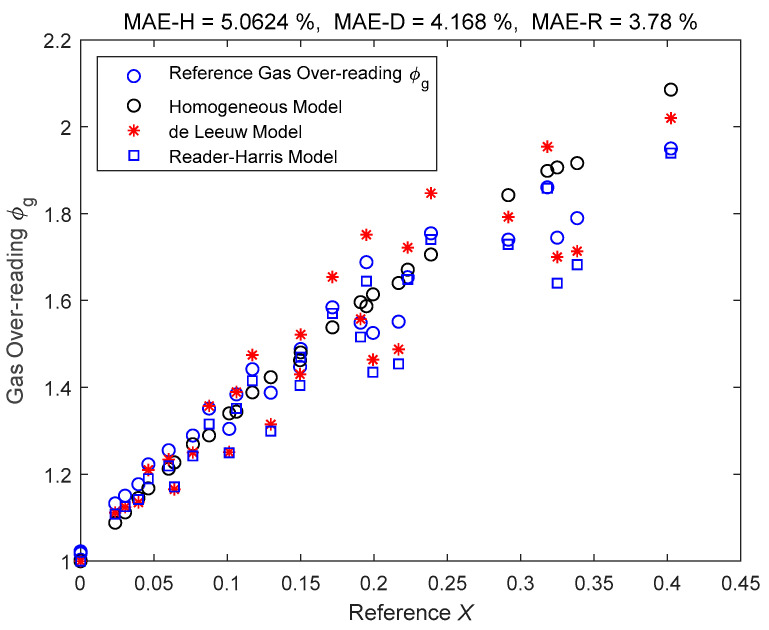
The prediction results of several common over-reading equations for the gas over-reading of an ETV.

Because $C_{d}\approx 1$ is assumed for other over-reading models, so the $\phi_{g}^{\mathrm{RH}}$ calculated by Equations (3) and (4) should be divided by $C_{d}$ to facilitate its comparison with other models. The data fitting results of the homogeneous model, de Leeuw model and Reader-Harris model are shown in Figure 4, where the MAE-H, MAE-D and MAE-R in the titles correspond to the MAE (defined in Equation (18)) of Homogeneous, de Leeuw and Reader-Harris models respectively.

From Figure 4, it can be noted that the data fitting results of Reader-Harris model (ISO/TR 11583 model) are slightly better than the de Leeuw model, whilst the data fitting results of the de Leeuw model are slightly better than the homogenous model. However, considering the complexity of the model, the number of parameters involved and the risk of overfitting, de Leeuw model is employed in this paper to calculate the gas over-reading $\phi_{g}$ and its initial value is provided by the homogeneous model.

#### 3.2.2. Determination of X

**Figure 5:**
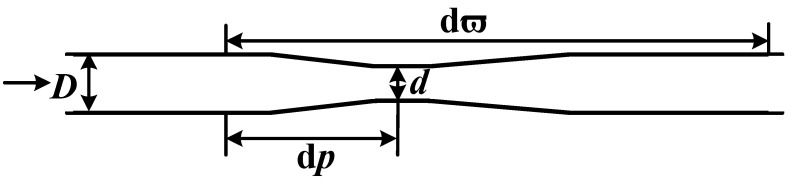
Schematic diagram of a classical Venturi.

The liquid content of wet gas is usually described by the L-M number $X=\frac{Q_{l}}{Q_{g}}\sqrt{\frac{\rho_{l}}{\rho_{g}}}$. According to the research by de Leeuw [24] and Reader-Harris [25,33], X is usually obtained from the data fitting with the pressure loss ratio $\frac{d\varpi }{dp}$, where dϖ represents the pressure loss across a classical Venturi tube, dp represents the acceleration pressure drop through the convergent section of Venturi, as shown in Figure 5. For the ETV, the above two parameters correspond to the rear differential pressure $dp_{2}$ and front differential pressure $dp_{1}$ respectively, as shown in Figure 1.

The pressure loss ratio should be normalized first:

(30) $$\frac{Y}{Y_{\max}}=\frac{\frac{\Delta p_{2}}{\Delta p_{1}}-{\frac{\Delta p_{2}}{\Delta p_{1}}|}_{\mathrm{dry}}}{{\left(\frac{\Delta p_{2}}{\Delta p_{1}}-{\frac{\Delta p_{2}}{\Delta p_{1}}|}_{\mathrm{dry}}\right)|}_{X=0.3}}$$

and then data fitted in the following form:

(31) $$\frac{Y}{Y_{\max}}=1-\exp\left[-aX^{c}\exp\left(-b\frac{Fr_{\mathrm{gas}}}{H}\right)\right]$$

For the classical Venturi tube, data fitting are carried out by Reader-Harris and the final form are embodied by the ISO/TR 11583 [2]:

(32) $$\frac{Y}{Y_{\max}}=1-\exp\left[-35X^{0.75}\exp\left(-0.28\frac{Fr_{\mathrm{gas}}}{H}\right)\right]$$

**Figure 6:**
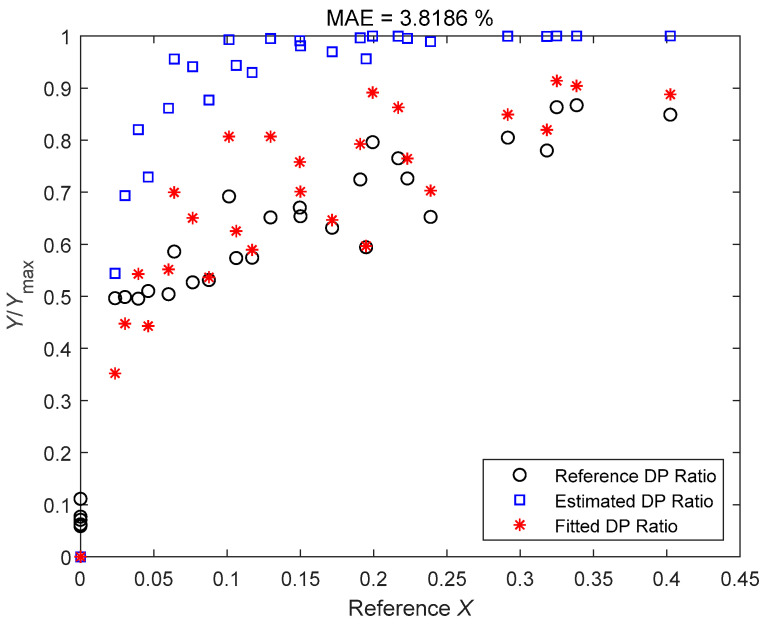
The estimates of the original and improved Reader-Harris correlation for the normalized differential pressure ratio of an ETV.

As there is obvious structure difference between the classical Venturi tube and the ETV, so if Equation (32) is applied to the ETV directly then serious deviations will occur, as shown in Figure 6. So, it is necessary to refit Equations (30) and (31) for the ETV and the results are as follows:

(33) $$\frac{Y}{Y_{\max}}=1-\exp\left[-5.5883X^{0.439}\exp\left(-0.2586Fr_{\mathrm{gas}}\right)\right]$$

where ${\frac{\Delta p_{2}}{\Delta p_{1}}|}_{\mathrm{dry}}$ and ${\frac{\Delta p_{2}}{\Delta p_{1}}|}_{X=0.4}$ are the minimum and maximum of the test data respectively, because the test data covered the two extreme cases of dry gas and X=0.4.

The fitting results of the improved Reader-Harris correlation for the normalized differential pressure ratio of an ETV are also shown in Figure 6, where the MAE (defined in Equation (18)) in the title refers to the MAE of the improved Reader-Harris correlation.

#### 3.2.3. Calculation Procedures

The iteration method combines the over-reading equation of the de Leeuw model (Equations (3), (4) and (27)) with the pressure loss ratio correlation of the Reader-Harris model (Equations (31) and (33)), and calculates the gas and liquid flowrate through iteration. As both the over-reading equation and the pressure loss ratio correlation require the gas Froude number $Fr_{g}$ to be known, this algorithm assumes $\hat{X}=0$ and uses the homogeneous model (Equations (3), (4) and (26)) for the initial values. The specific procedures of the iteration method are as follows:

1. First, the initial values of the L-M number $\hat{X}$ is assumed.
2. Then the gas over-reading ${\hat{\phi }}_{g}$ is estimated according to the homogenous model (Equation (26)).
3. Then the gas/liquid flowrates (${\hat{Q}}_{g}$ and ${\hat{Q}}_{l}$) are calculated from the definitions of gas over-reading ${\hat{\phi }}_{g}$ and L-M number $\hat{X}$ respectively.
4. Then ${\hat{Fr}}_{g}$ and ${\hat{Fr}}_{l}$ are calculated by its definition.
5. Then the gas over-reading ${\hat{\phi }}_{g}$ is calculated by the de Leeuw model (Equations (3), (4) and (27)). After ${\hat{\phi }}_{g}$ is calculated, ${\hat{\phi }}_{g}$ and $\hat{X}$ are substituted into the Step 3 to continue the following process until ${\hat{\phi }}_{g}$ converges.
6. Then the L-M number $\hat{X}$ is calculated by the pressure loss ratio correlation of the Reader-Harris model (Equations (30) and (33)). After $\hat{X}$ is calculated, it is then substituted into the Step 2 to continue the following process until $\hat{X}$ converges.

Finally, the last ${\hat{Q}}_{g}$ and ${\hat{Q}}_{l}$ are recorded and used as the estimates of the gas and liquid flowrates, respectively.

### 3.3. Comparison of the Direct Fitting and Iteration Method

**Figure 7:**
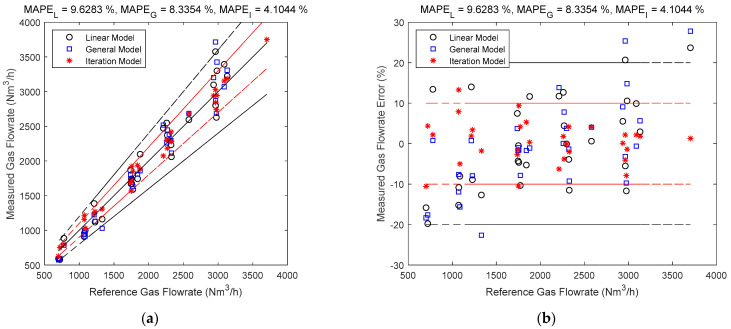
Gas flowrate estimates of the linear, general and iteration models: (**a**) absolute error; (**b**) relative error. (The central black line denotes the ideal case with zero error, the upper and lower red lines denote the 10% relative error range, and the upper and lower black lines denote the 20% relative error range. The vertical distance between the point and the central black line denote the error.

**Figure 8:**
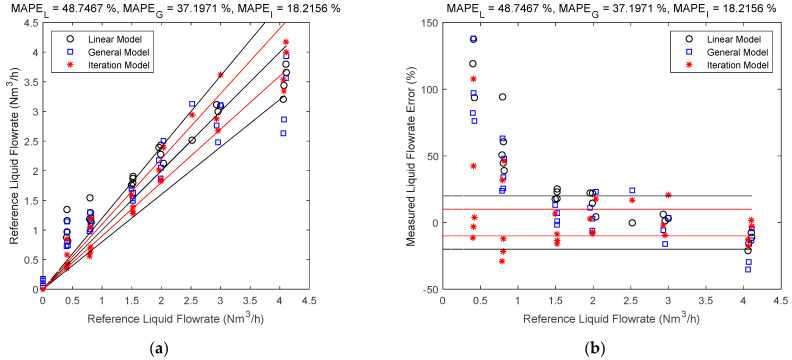
Liquid flowrate estimates of the linear, general and iteration models: (**a**) absolute error; (**b**) relative error. (The central black line denotes the ideal case with zero error, the upper and lower red lines denote the 10% relative error range, and the upper and lower black lines denote the 20% relative error range. The vertical distance between the point and the central black line denote the error.

The gas and liquid flowrate estimates of the linear, general and iteration models are shown in Figure 7 and Figure 8. The black circles denote the linear model results, the blue squares denote the general model results, and the red asterisks denote the iteration model results. The central black line denotes the ideal case when the estimated value is equal to the reference value so that the error is always zero. The upper and lower red lines specify a 10% relative error range so that points within this range have relative errors less than 10%. Similarly, the upper and lower black lines specify a 20% relative error range. The error is represented by the the vertical distance between the test point and the central black line. The ${\mathrm{MAPE}}_{L}$, ${\mathrm{MAPE}}_{G}$ and ${\mathrm{MAPE}}_{I}$ in the titles correspond to the MAPE (defined in Equation (19)) of linear, general and iteration models respectively.

**Figure 9:**
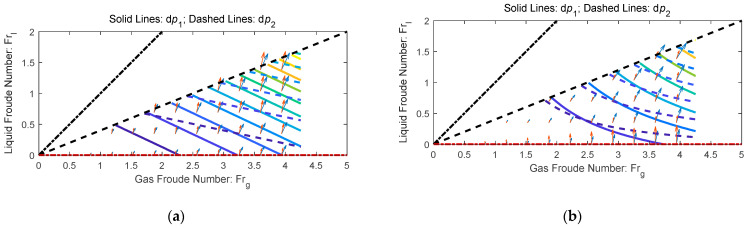
The comparisons of differential pressure (DP) contours of the linear model and the iteration model: (**a**) linear model; (**b**) iteration model.

It can be noted from Figure 7 and Figure 8 that the iteration model has the highest accuracy of the three methods, whilst for the direct fitting models, the general model is more accurate than the linear one. This is because as the models become more complicated, the impacts of more parameters are considered, and the contour maps of the differential pressures become closer to the real ones, as shown in Figure 9.

**Figure 10:**
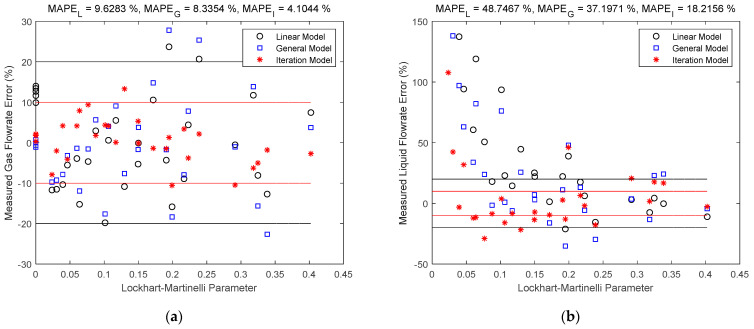
The relative errors of gas and liquid flowrates as a function of the Lockhart-Martinelli (L-M) number X: (**a**) gas flowrate; (**b**) liquid flowrate.

The relative errors of gas and liquid flowrates as a function of the L-M number X are shown in Figure 10a,b respectively, from which it can be noted that the relative errors of gas flowrate increase slowly with X, whilst the relative errors of liquid flowrate decrease rapidly with X. These results are generally in accord with the relative errors distributions of ETV, which will be shown in Section 4.2. It is worth mentioning that the influence of gas Froude number $Fr_{g}$ is not shown in Figure 10, and the trends revealed by the test results are subject to the randomness caused by the small numbers of samples.

## 4. Sensitivity Analysis and Error Ddistributions

### 4.1. Sensitivity Analysis

Although the iteration method can provide accurate results, it is relatively complicated and not very intuitive. Therefore, contour maps like Figure 9b can be used to facilitate our understanding. From Figure 9b, it can be noted that each contour line of $dp_{1}$ (solid line) and each contour line of $dp_{2}$ (dashed line) has only one intersection point, the x and y coordinates of which corresponds to the gas flowrate $Q_{g}$ (or $Fr_{g}$) and liquid flowrate $Q_{l}$ (or $Fr_{l}$) respectively, as long as the pressure p (or $\rho_{g}$) and geometric parameters does not change. Therefore, by looking for the intersection points of each $dp_{1}$ and $dp_{2}$ combination, we can easily find the corresponding $Fr_{l}$ and $Fr_{g}$ from Figure 9b, and then calculated the $Q_{l}$ and $Q_{g}$.

**Figure 11:**
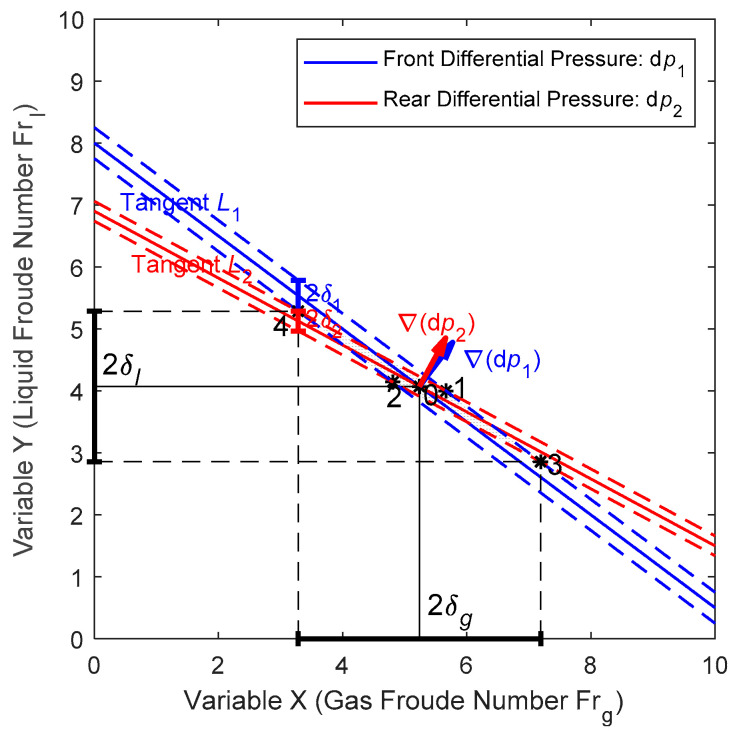
The method of estimating the relative errors of flowrates with the differential pressure contour maps.

In addition to calculating $Q_{l}$ and $Q_{g}$, the contour maps can also be used to estimate the relative error of the $Q_{l}$ and $Q_{g}$, and the working principle is shown in Figure 11. The blue solid line (tangent $L_{1}$) in Figure 11 represents the tangent of the contour of the front DP $dp_{1}$, whilst the red solid line (tangent $L_{2}$) represents the tangent of the contour of the rear DP $dp_{2}$. Therefore, Point 0 corresponds to the working condition and its coordinates correspond to the gas/liquid flowrates. As $dp_{1}$ and $dp_{2}$ are measured by the same type of pressure transmitter, then the relative error of $dp_{1}$ and $dp_{2}$ are assumed the same, which is σ=1% in this paper. The slopes of $L_{1}$ and $L_{2}$ are assumed to be $k_{1}$ and $k_{2}$ respectively, and it can be noted From Figure 11 that $\left|k_{1}\right|>\left|k_{2}\right|$.

From Figure 11, it can be clearly noted that if the measured differential pressure has an absolute error of $\delta \left(dp_{i}\right)=\sigma \cdot dp_{i}\;\left(i=\;1\;\mathrm{or}\;2\right)$, then it will cause an absolute error of $\delta_{g}$ and $\delta_{l}$ for the gas and liquid flowrate respectively. As the total derivative of differential pressure is $\delta \left(dp_{i}\right)=\nabla \left(dp_{i}\right)\cdot ds=\frac{\partial dp_{i}}{\partial x}dx+\frac{\partial dp_{i}}{\partial y}dy$, then the expression of $\delta_{i}$ (i=1 or 2) in Figure 11 can be written as: $\delta_{i}=\delta \left(dp_{i}\right)/\frac{\partial dp_{i}}{\partial y}$. Similarly, because the tangents and the gradients of the differential pressures $\nabla \left(dp_{i}\right)$ are mutually perpendicular, then the slope of the tangents can be expressed as:$k_{i}=\frac{\partial dp_{i}}{\partial x}/\frac{\partial dp_{i}}{\partial y}$.

From Figure 11, it can also be noted that $\delta_{g}$ and $\delta_{l}$ are mainly determined by Points 3 and 4, the coordinates of which are the solutions of two sets of binary functions. After some simplifications, the following expressions can be derived:

(34) $$\{\begin{array}{c} \delta_{g}=\frac{\delta_{1}+\delta_{2}}{k_{1}-k_{2}}\\ \delta_{l}=\frac{k_{2}\delta_{1}+k_{1}\delta_{2}}{k_{1}-k_{2}} \end{array}$$

After substituting the expressions of $k_{i}$ and $\delta_{i}$ into Equation (34) and introducing the definitions of relative errors $\sigma_{g}=\delta_{g}/Fr_{g}$, $\sigma_{l}=\delta_{l}/Fr_{l}$, we have:

(35) $$\{\begin{array}{c} \sigma_{g}=\frac{\sigma }{Fr_{g}}\frac{dp_{1}\frac{\partial dp_{2}}{\partial y}+dp_{2}\frac{\partial dp_{1}}{\partial y}}{\frac{\partial dp_{1}}{\partial x}\frac{\partial dp_{2}}{\partial y}-\frac{\partial dp_{2}}{\partial x}\frac{\partial dp_{1}}{\partial y}}\\ \sigma_{l}=\frac{\sigma }{Fr_{l}}\frac{dp_{1}\frac{\partial dp_{2}}{\partial x}+dp_{2}\frac{\partial dp_{1}}{\partial x}}{\frac{\partial dp_{1}}{\partial x}\frac{\partial dp_{2}}{\partial y}-\frac{\partial dp_{2}}{\partial x}\frac{\partial dp_{1}}{\partial y}} \end{array}$$

From Equation (35), it can be further derived that:

(36) $$\frac{\sigma_{l}}{\sigma_{g}}=\frac{1}{X}\frac{dp_{1}\frac{\partial dp_{2}}{\partial x}+dp_{2}\frac{\partial dp_{1}}{\partial x}}{dp_{1}\frac{\partial dp_{2}}{\partial y}+dp_{2}\frac{\partial dp_{1}}{\partial y}}\approx \frac{\left(\sqrt{\frac{\rho_{l}}{\rho_{g}}}+\sqrt{\frac{\rho_{g}}{\rho_{l}}}\right)+2/X}{\left(\sqrt{\frac{\rho_{l}}{\rho_{g}}}+\sqrt{\frac{\rho_{g}}{\rho_{l}}}\right)+2X}$$

From Equation (36), it can be noted that $\sigma_{l}/\sigma_{g}$ is only determined by the L-M number X, the $dp_{1}$ and $dp_{2}$, and their local gradients. The rightmost part of Equation (36) is based on the assumption that $\nabla \left(dp_{2}\right)\approx \nabla \left(dp_{1}\right)=\nabla \left(dp_{\mathrm{Hom}}\right)$, from which it can be noted that the ratio between the relative error of liquid and gas flowrate is a constant, which depends on the density ratio $\rho_{l}/\rho_{g}$ and the L-M number X.

### 4.2. Relative Error Distributions

**Figure 12:**
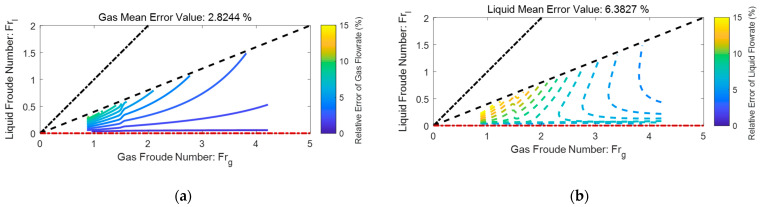
The relative error distributions of an ETV based on the $dp_{1}$ and $dp_{2}$ contour maps: (**a**) gas flowrate; (**b**) liquid flowrate.

The relative error distributions of an ETV tube are shown in Figure 12, where the color scale bar represents the local relative error of gas/liquid flowrate caused by 1% relative error of dp. The black dash-dotted line represents the L-M number of one, the black dashed line represents the L-M number of 0.4, whereas the red dash-dotted line represents the L-M number of zero. The results presented in the titles denote the mean value of relative errors of gas/liquid flowrates within the L-M range 0 to 0.4.

From Figure 12, it can be noted that 1% relative error of dp will cause roughly 2.8% of relative error for the gas flowrate and 6.4% of relative error for the liquid flowrate. In addition, the relative errors of gas and liquid flowrate generally increase with the L-M number X and decreases with the total flowrate $Q_{\mathrm{tot}}$, which suggests this device and algorithm are not very suitable for metering multiphase flow with high X or low $Q_{\mathrm{tot}}$. Under the same conditions, the liquid flowrate error is roughly 2~3 times the gas flowrate error, which is consistent with the predictions of Equation (36).

### 4.3. Comparison of the Classical and the ETV Tubes

**Figure 13:**
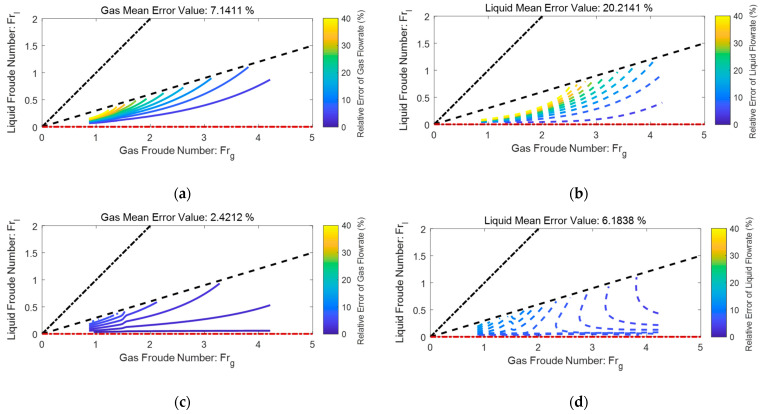
The comparison of the classical and the ETV: (**a**) the gas relative error distributions of the classical Venturi tube from the ISO/TR 11583; (**b**) the liquid relative error distributions of the classical Venturi tube from the ISO/TR 11583; (**c**) the gas relative error distributions of the ETV from the experiments; (**d**) the liquid relative error distributions of the ETV from the experiments.

By using a similar method, the contour maps of a classical Venturi tube can be generated from the empirical correlations provided in ISO/TR 11583 [2]. With the contour maps of the classical and ETV, it is possible to make a comparison between these two devices and the results are shown in Figure 13, where the color scale bar still represents the local relative error of gas/liquid flowrate caused by 1% relative error of dp. The black dashed line represents the L-M number of 0.3, and the results presented in the titles refer to the mean value of relative errors of gas/liquid flowrates within the L-M range 0 to 0.3. From Figure 13 it is notable that the ranges of X are both set as 0 to 0.3, and the relative error of all dp are set as 1% to make the comparison as fair as possible.

The relative error distributions of a classical Venturi tube are shown in Figure 13a, from which it can be noted that 1% relative error of dp will cause roughly 7.1% relative error for the gas flowrate and 20.2% relative error for the liquid flowrate. As a reference, the relative error distributions of an ETV are shown in Figure 13b, from which it can be noted that 1% relative error of dp will cause roughly 2.4% relative error for the gas flowrate and 6.2% relative error for the liquid flowrate. Therefore, it can be concluded that the impact of the dp error on the ETV is much less than the classical one, which suggests the ETV may be more accurate than the classical one under the same conditions. Meanwhile, the average velocity is much higher in the throat section of an ETV, which means the ETV can be designed to be more compact without undermining the signal intensity.

## 5. Conclusions

In this paper, theoretical models are established for the convergent and throat sections of an ETV. Several direct fitting and iteration flowrate algorithms are proposed and compared with the existing ones. The gradients of front and rear differential pressures are derived and the relationship between the differential pressure gradient and the flowrate relative error is studied analytically. Finally, the relative error distributions of an ETV are obtained and compared with the ones of a classical Venturi tube. The following important conclusions can be obtained through this research:

1. The iteration algorithm is more accurate than the direct fitting algorithm because it considers the influence of more parameters and therefore, its contour maps of differential pressures are closer to the reality than those of the other algorithms.
2. The gas flowrate error of the ETV iteration algorithm increases with the liquid content X whilst the liquid flowrate error of the ETV iteration algorithm decreases with the liquid content X.
3. The relative errors of liquid flowrates tend to be 2 to 3 times larger than those of the gas flowrates, which can be explained by the theoretical model and is in good agreement with the experimental results.
4. The ETV tube tends to be more accurate than the classical one. Additionally, it can be designed more compactly under the same signal intensity due to its significantly higher velocity in the throat section.

## Data Availability

The data presented in this study are available on request from the corresponding author.
